# BRASH Syndrome: A Patient With Chronic Kidney Disease and AV Nodal Blockers

**DOI:** 10.1155/crin/3405566

**Published:** 2025-05-12

**Authors:** Zein A Alsayed-Ahmad, Sanaa Saddour, Tasnim Mustafa Saddour, Mohamad Moafak Hariri, Karam Albitar, Sami Albitar

**Affiliations:** ^1^Faculty of Medicine, Aleppo University, Aleppo, Syria; ^2^Faculty of Medicine, Syrian Private University, Damascus, Syria

**Keywords:** atrioventricular blockade, bradycardia, BRASH syndrome, case report, hemodialysis, hyperkalemia, renal failure

## Abstract

**Background:** BRASH syndrome is a life-threatening condition that involves bradycardia, renal failure, atrioventricular blockade, shock, and hyperkalemia. It is often resistant to conventional treatments and requires prompt diagnosis and management. We report a case of BRASH syndrome successfully treated in the Emergency Department and Nephrology Department.

**Case Presentation:** A 57-year-old man with hypertension, diabetes, ischemic heart disease, and chronic kidney disease presented with severe diarrhea, lethargy, and shock. He had hyperkalemia, metabolic acidosis, and acute kidney injury. His electrocardiogram showed sinus bradycardia with complete AV block. He was on bisoprolol, which was discontinued. He received hemodialysis, potassium-lowering agents, and vasoactive drugs. His renal function improved, and his heart rate normalized with first-degree AV block. He was discharged with advice to avoid AV-blocking agents and follow-up with nephrology and cardiology.

**Conclusions:** BRASH syndrome is a serious complication of hyperkalemia, hypotension, and bradycardia in patients with kidney dysfunction and AV-blocking medications. It may require hemodynamic support and temporary pacemaker insertion. Early recognition and treatment of this entity can reduce mortality and morbidity.

## 1. Introduction

Atrioventricular (AV) nodal-blocking agents in patients with chronic kidney disease (CKD) can result in worsening of renal function and hyperkalemia, which in turn can lead to bradycardia. This results in low cardiac output, which induces renal hypoperfusion and dysfunction, followed by shock. Kidney injury can perpetuate hyperkalemia. This pathophysiological vicious cycle was recently recognized in 2016 as BRASH syndrome, an acronym that refers to the pentad of bradycardia, renal failure, AV blockade, shock, and hyperkalemia. To date, only approximately 70 cases have been reported [1, 2]. Despite its rarity, BRASH syndrome is a potentially fatal condition that lacks evidence-based guidelines for its management, and likely under-reported due to its relatively recent recognition and complex physiopathology [3].

Patients with CKD are at heightened risk for BRASH syndrome due to compromised potassium excretion, susceptibility to renal ischemia, polypharmacy, and prevalent cardiovascular disease. These conditions make them vulnerable to the effects of AV nodal blockers.

We report a case of a 57-year-old man with CKD who developed BRASH syndrome after taking bisoprolol, a beta blocker. He was successfully treated with a combination of pharmacological and dialytic interventions that restored his hemodynamic stability and renal function. Our case demonstrates the importance of prompt recognition and treatment of BRASH syndrome in patients with CKD who are taking AV nodal-blocking agents.

## 2. Case Presentation

A 57-year-old man with a medical history of CKD Stage 4 (creatinine clearance of 15.37 mL/min/m^2^), type 2 diabetes mellitus, ischemic heart disease, and hypertension regularly taking aspirin, clopidogrel, and bisoprolol 5 mg once daily, in addition to spironolactone, furosemide, febuxostat, trimetazidine, sacubitril, valsartan, empagliflozin, and linagliptin.

He was admitted to the Emergency Department with complaints of weakness and shortness of breath, which had worsened over the few hours before his admission.

Upon arrival, the patient was initially alert with a Glasgow Coma Scale (GCS) of 15, indicating full consciousness. His initial vital signs were within normal limits, showing no immediate signs of distress, and the patient denied angina or palpitations. However, shortly after the initial assessment, the patient's condition rapidly deteriorated into shock and syncope.

The patient presented with mottled skin, reduced urinary output (12.5 mL/h), and lactate levels of 1.1 mmol/L, indicating a shock state.

He experienced a decrease in the level of consciousness, with a GCS score of 5/15, associated with sustained bradycardia.

Upon presentation, the patient had a heart rate of 28 beats per minute and a blood pressure of 60/40 mmHg (mean arterial pressure of 53 mmHg) and a body temperature of 36.6°C. The respiratory rate was 18 breaths per minute, and peripheral oxygen saturation was 98% without supplementary oxygen.

His physical examination was otherwise unremarkable. Laboratory results were notable for a white blood cell count of 5800 cells/μL, creatinine level of 7.5 mg/dL, serum potassium level of 6.3 mg/dL, ABG: ph: 7.226, hco3: 19.3 mmol/L, pco2: 48.2 mmHg, po2: 112 mmHg, and glucose fasting: 210 mg/dL (Table 1).

**Figure 1:**
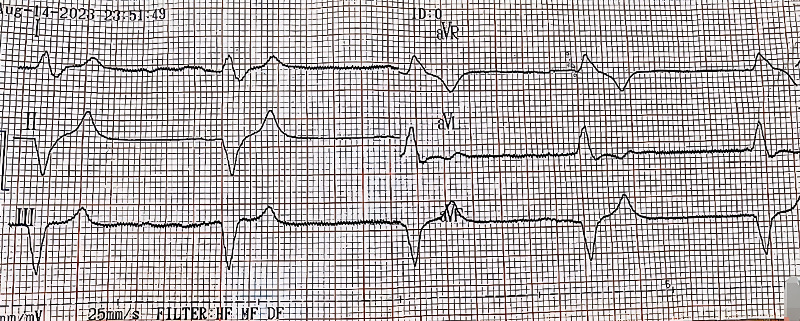
Admission ECG showing sinus bradycardia with complete AV block.

The initial electrocardiogram (ECG) (Figure 1) showed idioventricular rhythm with a heart rate of 38 bpm. P waves were absent. A chest X-ray was normal. Transthoracic echocardiography showed normal systolic and diastolic function, no valvular abnormalities, and a left ventricular ejection fraction of 57%. Differential diagnoses considered included BRASH syndrome, cardiogenic shock due to bradyarrhythmia, acute kidney injury, and mild hypercalcemia.

An intravenous (i.v.) bolus of 20 mL/kg 0.9% sodium chloride was started. Four doses of 0.5 mg of i.v. atropine were administered, in addition to 20 mL of 10% calcium gluconate, 100 mg of hydrocortisone, 70 mL of sodium bicarbonate, and a solution of 250 mL of 20% dextrose with 10 U of regular insulin. Previous medications were suspended. Blood pressure was maintained at < 65 mmHg despite initial measures, so a continuous infusion of dopamine at 15 mcg/kg/min was initiated, primarily for its beta-adrenergic effects. The patient was admitted to the intensive care unit (ICU) for early hemodialysis within the first few hours. His hyperkalemia was corrected on the first day of admission, and creatinine levels progressively returned to baseline values. On Day 2, hemodynamic stability was achieved after correction of hyperkalemia and renal function. On Day 4, the patient's vitals normalized, and he was discharged for outpatient nephrology and cardiology follow-up, with the recommendation to discontinue beta-blockers (bisoprolol) and continue his other prescribed medications, which collectively support his multifaceted medical needs.

**Figure 2:**
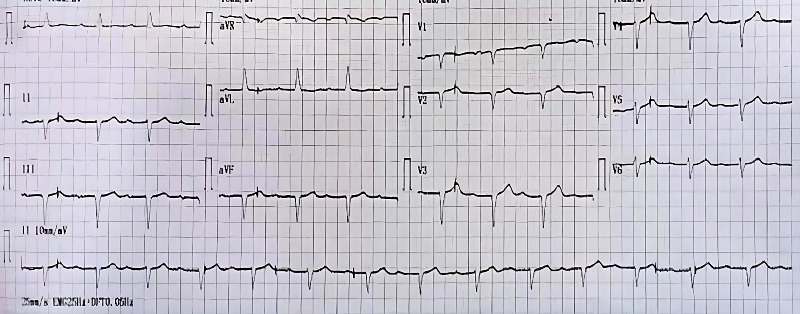
Discharge ECG showing first-degree block.

Upon discharge, ECG showed a first-degree block (Figure 2), serum creatinine was 3 mg/dL, and potassium was 4.5 mg/dL (Table 1).

## 3. Discussion and Conclusion

BRASH syndrome is a rare but potentially life-threatening condition involving bradycardia, renal failure, AV-nodal blockade, shock, and hyperkalemia [4].

While typically diagnosed in patients presenting with bradycardia in the setting of a combined effect of hyperkalemia and a history of use of AV-nodal blockers, along with a medical condition that causes renal insufficiency [5], some argue it may represent a specific syndrome with a characteristic pathophysiology. So, more research and evidence are needed to clarify the nature, prevalence, and management of BRASH syndrome.

The most common symptoms of BRASH syndrome according to a systematic review of the literature conducted by Majeed et al. are fatigue, somnolence, encephalopathy, and syncope, which reflect decreased cerebral perfusion. However, some cases reported dyspnea and diarrhea as symptoms, similar to our case [1]. One possible explanation is that diarrhea may cause mild dehydration that can trigger the vicious cycle; for example, a case presented in summer months with severe bradyarrhythmia and needed transvenous pacing, which was likely induced by dehydration [6].

Diagnosing BRASH syndrome can be challenging due to overlapping factors with other conditions. The most common differential diagnoses are isolated hyperkalemia and intoxication with AV-nodal blocking agents. First, in contrast to isolated hyperkalemia, BRASH syndrome can provoke bradycardia even with lower potassium levels (with a mean serum concentration at presentation of 5.2) [1]. Additionally, the ECG may not exhibit typical hyperkalemia indicators such as peaked T waves or sine waves.

On the other hand, to differentiate it from intoxication with AV-nodal blocking agents, BRASH syndrome patients typically adhere to their medication as prescribed and do not have excessively high drug levels. Instead, the problem arises from the synergy between therapeutic drug levels and hyperkalemia [2].

While advanced cardiovascular life support (ACLS) principles, such as dopamine administration, are useful, BRASH syndrome necessitates individualized care due to the complex interplay of hyperkalemia and bradycardia [5].

The management strategy is based on three aspects: (1) correcting hyperkalemia, (2) providing hemodynamic support for bradycardia and hypotension, and (3) treating triggering events (e.g., hypovolemia, AV-nodal block medication ischemia, inflammation, infection, or trauma) [7].

The first step is to withdraw triggering agents (bisoprolol in our case) [8].

Second, IV saline along with atropine and dopamine was used to manage the hemodynamic state, even though a study by Farkas et al. suggested that the use of isoproterenol has a much more profound chronotropic effect and is the agent of choice when hypotension is the result of bradycardia [2]. However, it was not available for us to use at the time.

Moreover, since managing hyperkalemia is a priority, administration of IV calcium was advised to stabilize the cardiac membrane and improve cardiac output; however, it does not reduce the extracellular potassium concentration [8]. Intravenous insulin and dextrose can be given to shift potassium intracellularly. Albuterol is another option to be considered, with potential benefits in terms of both hyperkalemia and bradycardia.

Dopamine was used in this case due to its beta-adrenergic effects, increasing heart rate and cardiac output to counter bradycardia and hypotension. An alternative could have been dobutamine, a selective beta-1 agonist, which enhances myocardial contractility and is often used in cardiogenic shock. However, dopamine was effective and readily available in our setting.

Considering that our patient went rapidly into shock, emergent short-term dialysis was needed as a definitive treatment for hyperkalemia. Usually, a coordinated treatment approach to BRASH can avoid dialysis, but our patients have already progressed to anuric renal failure. Typically, dialysis can reverse hyperkalemia before temporary pacing is necessary. Additionally, 100 mg IV hydrocortisone was given as a stress dose to manage adrenal insufficiency [2].

In conclusion, BRASH syndrome is a life-threatening condition that is often overlooked. The interaction between AV-nodal–blocking agents and hyperkalemia may be involved in its pathophysiology, but more research is needed to clarify the mechanism and establish guidelines. Early recognition and intervention are essential to prevent the development of multiple-organ failure and death.

## Figures and Tables

**Table 1:** Laboratory values from baseline, admission, and discharge.

	Baseline	Admission	Discharge	Reference value
Creatinine (mg/dL)	3.33	7.5	3	0.55–1.02

Creatinine clearance (mL/min/m^2^)	—	15.37	38.43	62–139

Potassium (mEq/L)	5.1	6.3	4.5	3.5–5.0

Sodium (mEq/L)	137	122	134	136–145

Glucose fasting	155	210	135	90–130

WBC (/μL)	6370	5800	6380	3500–10,500

Hb (g/dL)	9.3	9	9.5	12.0–15.5

Pt (second)	—	16.2	12.3	Control: 13.5

Bicarbonate (mmol/L)	—	19.3	22.7	20.0–24.0

Lactate (mmol/L)	—	1.10	1	Less than 1

INR	—	1.2	1.1	1–1.3

ABG: pH	—	pH: 7.226	pH: 7.356	pH: 7.35–7.45
Hco3		hco3: 19.3 mmol/L	hco3: 22.3 mmol/L	Hco3: 22–26 meq/L
pco2		pco2: 48.2 mmhg	pco2: 44.5 mmhg	Pco2: 35–45 mmhg
po2		po2: 112 mmhg	po2: 98 mmhg	Po2: 75–100 mmhg

*Note:* Hb, hemoglobin; WBC, white blood cell count.

Abbreviations: ABG, arterial blood gases; INR, international normalized ratio; Pt, prothrombin time.

## Data Availability

All data generated or analyzed during this study are included in this published article.

## Nomenclature

BRASH syndrome: Bradycardia, renal failure, atrioventricular blockade, shock, and hyperkalemia
AV: Atrioventricular
ECG: Electrocardiogram
ICU: Intensive care unit
IV: Intravenous
ACLS: Advanced cardiovascular life support
